# Editorial: Cytotoxic CD4+ T Cells in Viral Infections

**DOI:** 10.3389/fimmu.2017.01729

**Published:** 2017-12-04

**Authors:** Chansavath Phetsouphanh, Shiv Pillai, John J. Zaunders

**Affiliations:** ^1^Peter Medawar Building for Pathogen Research, Nuffield Department of Medicine, University of Oxford, Oxford, United Kingdom; ^2^Massachusetts General Hospital, Harvard Medical School, Boston, MA, United States; ^3^The Kirby Institute, University of New South Wales, Sydney, NSW, Australia; ^4^St Vincent’s Centre for Applied Medical Research, St Vincent’s Hospital, Sydney, NSW, Australia

**Keywords:** T cells, immunology, viral infections, CD4 T cells, cytotoxic CD4 T cells

**Editorial on the Research Topic**

**Cytotoxic CD4+ T Cells in Viral Infections**

Since the 1970s, recurrent observations have been made that CD4+ T cells are not mere helpers but can also have cytotoxic activity, as originally described in murine allogeneic responses and then in human memory CD4 T cell recall responses to viral antigens. Pathogens that chronically infect cells of the human immune system, including some of the world’s most prevalent persistent infections, such as EBV, CMV, HIV-1, TB, HHV-8, and HTLV-1, are usually thought of as controlled by CD8 CTL and neutralizing antibodies, but these pathogens have evolved mechanisms to avoid this control, especially downregulation of HLA Class I antigen presentation. However, the target cells of these pathogens constitutively express HLA Class II and therefore represent a potentially direct focus of CD4 effector cells. In fact, a recent study showed that even CD8 T cells may use HLA Class II restriction to control CMV infection in a rhesus macaque model (1).

*In vivo* murine models have recently confirmed that cytotoxic CD4+ T cells (CD4 CTL) are a unique CD4 T cell subset with cytolytic capabilities. In the context of viral infections such as West Nile virus (2), influenza (3), and Friend virus (4), it has been demonstrated that cytotoxic CD4+ T cells are readily detectable *ex vivo* and can contribute to viral containment even in the absence of antigen-specific CD8+ T cell or B cell responses. Moreover, it has also been shown that cytotoxic CD4+ T cells may play an important protective role in chronic viral infections, as evidenced by their control of viral replication in EBV and CMV infection. CD4+ T cells from HIV-infected patients have been shown to express large quantities of cytolytic effector molecules such as perforin and granzymes, immediately *ex vivo*, and HIV-specific CD4+ T cell clones and cell lines can readily mediate target cell lysis and viral inhibition *in vitro* (5). Distinct phenotype, lineage commitment, and function of these cytotoxic CD4+ T cells in viral infections are currently under intense investigation.

Based on studies on virus-specific T cells in humans, it was postulated that effector memory T cells differentiate in a linear path from CD28+CD27+ (early-differentiated) to CD28+CD27− (intermediate-differentiated) to CD28−CD27− (late-differentiated) (6). Presence of CD4+CD28negative or null T cells has been extensively observed in inflammation and auto-immunity for the past 20 years. They have distinct regulatory pathways during activation and have been postulated to be replication senescent (7). Chronic CMV infection drives the CD4+CD28− phenotype, and their expansion is associated with cardiovascular disease, rheumatoid arthritis, and other autoimmune conditions (Broadley et al.). CD4+CD28− cells produce large amounts of IFNγ and TNFα, express cytotoxic granule markers granzyme B and perforin, and exert cytolytic activity demonstrated by *in vitro* and *in vivo* models (Muraro et al.). Virus-driven expansion of these cytotoxic cells may cause complications to autoimmunity, where they play an effector role that mediates inflammation. However, current studies demonstrate that these very same cells may have a protective role against viral infections. They have presumably evolved to offer protections against viral infections and cancer (8) but can also potentially represent a T cell subset that drives inflammation and fibrosis.

The role of CD4 CTL on the containment of viral replication has often been overshadowed by the presence of cytotoxic CD8+ T cells that outnumber them. CD8+ T cells have long been recognized as the major contributor of control in acute and chronic viral infections; however, their role is now not as substantial as previously thought. Incomplete control and viral immune escape mechanisms have been described together with the ability of some viruses to downregulate MHC class I expression, which suggests that an alternate cytotoxic pathway may contribute to viral control. CD4 CTL that recognizes viral epitopes via MHC class II may act in concert with CD8 CTL to further strengthen viral control (Muraro et al.). CD4 CTL has been shown to play a pivotal role in the containment of viral replication in influenza, EBV, and CMV infections (Juno et al.). Recent studies on HIV-1, dengue virus (Tian et al.), and HPV have shown that CD4 CTL cells are activated and highly cytotoxic and may prevent disease progression.

Markers specific for CD4 CTL have not been identified. Whether CD4 CTL composed of a single subset or multiple subtypes requires further examination. Innate activation markers, such as NKG2D and KLRG1, have been identified on their surface. Recently, Takeuchi and Saito identified the class I-restricted T cell-associated molecule (CRTAM) as a marker of CD4 CTL (9). CRTAM is an early activation marker of NK and CD8+ T cells. CRTAM is expressed transiently on early-activated CD4+ T cells that have cytotoxic activity. However, due to the kinetics of CRTAM expression, its use as an *in vivo* CD4 CTL marker is limited. Mattoo et al. identified SLAMF7 as a marker of CD4 CTL in IgG4-related disease; a chronic inflammatory syndrome that can affect any organ system in the body and manifests as storiform fibrosis, obliterative phlebitis, tumefactive lesions, and high levels of IgG4-secreting plasma cells in inflamed tissues. CD4+ T cells that express SLAMF7 displayed a cytotoxic phenotype with the expression of Granzyme A, IL-1β, and TGF-β1 and were clonally expanded in inflamed IgG4-RD tissue sites (10). SLAMF7 represents a marker that distinguishes CD4+CTLs from all other CD4+ T cell subsets.

Limited studies on the differentiation and proliferation of CD4+CTLs have been reported. CD4 CTLs can apparently develop from multiple T helper subsets, with Th1-like cells representing the majority of CD4 CTL expressing IFNγ, TNFα, and/or IL-2 (5, 11), but also CD40L (12). CD4 CTL differentiation and function, such as other T helper subsets, require essential transcription factors that orchestrate transcription of key genes. Tbet and Eomes are important CD8 transcription factors that have been postulated to regulate CD4 CTL function, and the level of expression of Tbet and Eomes may depend on their maturation stage (Brown et al.). Recently, Oja et al. identified that the transcription factor Hobit (Homologo of Blimp-1 in T cells) was specifically upregulated in CD45RA+ effector CD8+ T cells in hCMV infection. Hobit expression was further identified in CD4+ T cells, and Hobit+ CD4+ T cells had a cytotoxic profile similar to Hobit+ CD8+ T cells, including the expression of Tbet and fractalkine receptor CX3CR1 (Oja et al.). It is still unclear however, what signals are required for the upregulation of Hobit after hCMV infection. Tbet is postulated to be responsible for the maintenance of Hobit after viral clearance, although it has not been proven. Whether Hobit is also upregulated in other viral infections and its role in CD4 CTL function warrants further examination.

CD4 CTL cells are long-lived and have been shown in many viral infections to be effective in the elimination of infected target cells. With these properties, CD4 CTL cells are thus a potential candidate for adoptive transfer strategies for cellular treatment of chronic viral infections and virus-driven tumors. However, several hurdles need to be overcome to fully utilize these cells for therapy. Identification of lineage markers and transcription factors need to be defined, as well as their expansion and maintenance (essential homeostatic cytokines) needs to be determined. Emerging research on this subset will expand our knowledge of the complex T helper response and will shed light on another effector cell type that has a killing potential.

## Author Contributions

CP wrote the manuscript, SP and JZ edited and finessed the manuscript.

## Conflict of Interest Statement

The authors declare that the research was conducted in the absence of any commercial or financial relationships that could be construed as a potential conflict of interest.

## References

[1] Ranasinghe S Lamothe P Damien S Jones B Sidney J Sette A Paradigm-violating HLA class II-restricted CD8 T cells exist in HIV infected individuals (VIR6P.1164). J Immunol (2015) 194:149.4.

[2] Brien JD Uhrlaub JL Nikolich-Žugich J. West Nile virus-specific CD4 T cells exhibit direct antiviral cytokine secretion and cytotoxicity and are sufficient for antiviral protection. J Immunol (2008) 181(12):8568. 10.4049/jimmunol.181.12.8568 19050276 PMC3504655

[3] Brown DM Lee S Garcia-Hernandez Mde L Swain SL. Multifunctional CD4 cells expressing gamma interferon and perforin mediate protection against lethal influenza virus infection. J Virol (2012) 86(12):6792–803. 10.1128/JVI.07172-11 22491469 PMC3393557

[4] Manzke N Akhmetzyanova I Hasenkrug KJ Trilling M Zelinskyy G Dittmer U. CD4+ T cells develop antiretroviral cytotoxic activity in the absence of regulatory T cells and CD8+ T cells. J Virol (2013) 87(11):6306–13. 10.1128/JVI.00432-13 23536666 PMC3648127

[5] Appay V Zaunders JJ Papagno L Sutton J Jaramillo A Waters A Characterization of CD4+; CTLs ex vivo. J Immunol (2002) 168(11):5954 10.4049/jimmunol.168.11.5954 12023402

[6] Appay V The physiological role of cytotoxic CD4(+) T-cells: the holy grail? Clin Exp Immunol (2004) 138(1):10–3. 10.1111/j.1365-2249.2004.02605.x 15373899 PMC1809194

[7] Vallejo AN Brandes JC Weyand CM Goronzy JJ. Modulation of CD28 expression: distinct regulatory pathways during activation and replicative senescence. J Immunol (1999) 162(11):6572. 10352273

[8] Malandro N Budhu S Kuhn Nicholas F Liu C Murphy Judith T Cortez C Clonal abundance of tumor-specific CD4+ T cells potentiates efficacy and alters susceptibility to exhaustion. Immunity (2016) 44(1):179–93. 10.1016/j.immuni.2015.12.018 26789923 PMC4996670

[9] Takeuchi A Badr MESG Miyauchi K Ishihara C Onishi R Guo Z CRTAM determines the CD4+ cytotoxic T lymphocyte lineage. J Exp Med (2016) 213(1):123. 10.1084/jem.20150519 26694968 PMC4710199

[10] Mattoo H Mahajan VS Maehara T Deshpande V Della-Torre E Wallace ZS Clonal expansion of CD4+ cytotoxic T lymphocytes in patients with IgG4-related disease. J Allergy ClinImmunol (2016) 138(3):825–38. 10.1016/j.jaci.2015.12.1330 PMC5014627 26971690

[11] van Leeuwen EMM Remmerswaal EBM Vossen MTM Rowshani AT Wertheim-van Dillen PME van Lier RAW Emergence of CD4+CD28-Granzyme B+, cytomegalovirus-specific T cell subset after recovery of primary cytomegalovirus infection. J Immunol (2004) 173(3):1834 10.4049/jimmunol.173.3.1834 15265915

[12] Zaunders JJ Dyer WB Wang B Munier ML Miranda-Saksena M Newton R Identification of circulating antigen-specific CD4+ T lymphocytes with a CCR5+, cytotoxic phenotype in an HIV-1 long-term nonprogressor and in CMV infection. Blood (2004) 103(6):2238. 10.1182/blood-2003-08-2765 14645006

